# Biological Characterization of Gene Response to Insulin-Induced Hypoglycemia in Mouse Retina

**DOI:** 10.1371/journal.pone.0150266

**Published:** 2016-02-26

**Authors:** Martine Emery, Natacha Nanchen, Frédéric Preitner, Mark Ibberson, Raphaël Roduit

**Affiliations:** 1 IRO, Institute for Research in Ophthalmology, Sion, Switzerland; 2 Mouse Metabolic Evaluation Facility, Center for Integrative Genomics, University of Lausanne, Lausanne, Switzerland; 3 Vital-IT Group, Swiss Institute of Bioinformatics, University of Lausanne, Lausanne, Switzerland; 4 Department of Ophthalmology, University of Lausanne, Jules-Gonin Eye Hospital, Fondation Asile des Aveugles, Lausanne, Switzerland; Universidade Federal do ABC, BRAZIL

## Abstract

Glucose is the most important metabolic substrate of the retina and maintenance of normoglycemia is an essential challenge for diabetic patients. Chronic, exaggerated, glycemic excursions could lead to cardiovascular diseases, nephropathy, neuropathy and retinopathy. We recently showed that hypoglycemia induced retinal cell death in mouse via caspase 3 activation and glutathione (GSH) decrease. *Ex vivo* experiments in 661W photoreceptor cells confirmed the low-glucose induction of death via superoxide production and activation of caspase 3, which was concomitant with a decrease of GSH content. We evaluate herein retinal gene expression 4 h and 48 h after insulin-induced hypoglycemia. Microarray analysis demonstrated clusters of genes whose expression was modified by hypoglycemia and we discuss the potential implication of those genes in retinal cell death. In addition, we identify by gene set enrichment analysis, three important pathways, including lysosomal function, GSH metabolism and apoptotic pathways. Then we tested the effect of recurrent hypoglycemia (three successive 4h periods of hypoglycemia spaced by 48 h recovery) on retinal cell death. Interestingly, exposure to multiple hypoglycemic events prevented GSH decrease and retinal cell death, or adapted the retina to external stress by restoring GSH level comparable to control situation. We hypothesize that scavenger GSH is a key compound in this apoptotic process, and maintaining “normal” GSH level, as well as a strict glycemic control, represents a therapeutic challenge in order to avoid side effects of diabetes, especially diabetic retinopathy.

## Introduction

Microvascular complications leading to nephropathy, cardiovascular diseases, neuropathy and retinopathy are serious consequences of both type I and II diabetes [1]. Diabetic retinopathy is generally linked to hyperglycemia [2], but recently we [3–5] and other [6–10] have described the key role of hypoglycemia in retinal visual dysfunction and cell death. There are several different causes of hypoglycemia, including inappropriate diet, irregular physical activity and unsuitable insulin treatment of diabetes. This latter, called iatrogenic hypoglycemia, causes cerebral edema leading to coma, even mortality, in people with type I diabetes and in many with advanced type II diabetes [11]. In addition, retinopathy of prematurity may also be the consequence of hypoglycemic periods that alter brain (and retina) development [12]. Very little is known about the real effect of chronic or acute hypoglycemic event on visual functions.

The majority of studies have focused on cellular models showing that low glucose conditions reduced viability of all retinal cell types in a mixed primary cell culture [13], while aglycemic conditions forced isolated chick retinas to metabolize and use lactate to preserve ATP levels and retinal neuronal cell death [14]. Diverse *in vivo* studies showed that chronic moderate hypoglycemia observed in glucagon receptor-deficient mice (Gcgr^-/-^) led to loss of vision and possible retinal degeneration [6], long-term exposure of this mouse model to carbohydrate diet normalized glycemia and partially restored retinal function [9]. In human, acute effect of hypoglycemia involve principally central vision [8]. More generally, Punzo *et al*. recently suggested that cone death in retinitis pigmentosa could be, at least in part, the result of the starvation of cones via the insulin/mTOR pathway [7]. We previously showed that insulin-induced hypoglycemia led to retinal cell death, possibly through a modification of glutathione (GSH) metabolism that alters GSH level of the cell [3]. We further detailed the mechanism of apoptosis involved in this acute hypoglycemic mouse model and described the role of Bcl-2/Bax in the process. Furthermore, we define an important role of autophagy in the protection of low glucose-induced 661W photoreceptor cell death [4].

Strict monitoring and maintenance of normoglycemia is a key element for diabetic patients in order to minimize both hyper- and hypoglycemic events. Avoiding multiple hypoglycemic events is even more critical because recurrent episodes of hypoglycemia lead to hypoglycemia-associated autonomic failure (HAAF). In this regard, repeated hypoglycemia leads to a defect in hypoglycemia-induced activation of counter-regulatory mechanisms (namely, an attenuation of the normal epinephrine response to hypoglycemia, whilst insulin is not reduced and glucagon not increased by low glucose). Repeated hypoglycemia also causes hypoglycemia unawareness, which leads to the degradation of glycemic control by patients. Thus, in this vicious cycle HAAF increases the risk of recurrent and severe hypoglycemic events because of the inability to recognize hypoglycemia [15, 16].

In this study, we sought to characterize the changes in gene expression in the whole retina of mice undergoing acute hypoglycemia during 4 hours with a recovery time ranging from 4 hours to 2 days. We performed Affymetrix microarray analysis in mouse retinas isolated after a hyperinsulinemic/hypoglycemic or hyperinsulinemic/euglycemic clamp. We highlighted diverse pathways involved in apoptosis, lysosomes and glutathione metabolism. Moreover, we performed multiple hyperinsulinemic/hypoglycemic or hyperinsulinemic/euglycemic clamps to test whether this treatment, often called preconditioning, could more strongly impact or protect retinal cell death. Interestingly, no cell death was observed after multiple successive clamps, suggesting a protective effect or an adaptation to hypoglycemic events.

## Methods

### Mouse line

This study adhered to the Association for Research in Vision and Ophthalmology (ARVO) statement for the use of animals in ophthalmic and vision research and was approved by the Veterinary service of the State of Valais (Switzerland). Three month-old C57BL/6J female mice were purchased from Charles River Laboratories (Les Oncins, France). Animals were kept at 23°C in a 12-h light/12-h dark cycle (7am-7pm) with unlimited access to chow diet (3436, Provimi Kliba AG, Kaiseraugst, Switzerland) and water in vented, filtered M.I.C.E. cages (Animal Care Systems Inc, CO, USA) in the housing room of the Mouse Metabolic Facility, Center for Integrative Genomics, University of Lausanne, Lausanne, Switzerland) with conventional-experimental health status (no MHV).

### Hyperinsulinemic clamps

Hyperinsulinemic clamps were performed as previously described [3] in 25–30g mice. An indwelling catheter (Becton Dickinson AG, Basel, Switzerland) was inserted into the femoral vein in isoflurane-anesthetized mice. Pediatric Dafalgan was administered in drinking water (2mg/ml) 1 day prior, and 2 days post-operation. Mice were allowed to recover for 7 days, after which they had recovered 98–110% of their pre-operative body weight.

Experiment 1 (single clamps): After a 5-h fast (7:30–12:30am), awake and freely moving mice were randomized and subjected to 4 h of either a hyperinsulinemic/hypoglycemic (sHypo, n = 16) or hyperinsulinemic/euglycemic (sEugly, n = 18) clamp as described [17]. The clamp was initiated by prime-infusion of human insulin (Actrapid, Novo Nordisk, Denmark, 18 mU/kg/min), and 50% glucose was infused at variable rates and adjusted to clamp plasma glucose around 2mM (sHypo) or 6mM (sEugly) as measured by glucometers on 2ul blood samples (Ascensia Breeze2, Bayer Healthcare, Switzerland) every 10 min during the stabilization period and every 20 minutes thereafter in order to minimize bleed volume (total 55–60ul). After 4 hours of clamp, insulin infusion was stopped and glucose infusion was maintained until the return to euglycemia was warranted in all groups. Mice were then returned to their cages with free access to food and water. Mice were sacrificed 4 or 48 h post-clamp by CO_2_ inhalation followed by decapitation and isolated retinas were used to prepare mRNAs for microarray and others analysis (S1 Table).

Experiment 2 (multiple and single clamps): After a 5-h fast (7:30–12:30am), awake and freely moving mice were randomized and subjected to three consecutive clamps performed in the same mice as described above, with a 2 days recovery after each hyperinsulinemic/hypoglycemic (mHypo, n = 6) or hyperinsulinemic/euglycemic (mEugly, n = 4) clamps. During the last clamp, two additional groups of mice were subjected to either a single hyperinsulinemic/hypoglycemic (sHypo, n = 6) or a single hyperinsulinemic/euglycemic (sEugly, n = 4) clamps. All mice were sacrificed 48 h post-clamp and isolated retinas were used to prepare retinal flat-mount for TUNEL assay, mRNAs for qPCR analysis, protein lysates for GSH measurement. In addition whole eyes were used for immunohistochemistry analysis (S2 Table).

### Microarray analysis

RNA samples isolated from retina, 4 and 48 h after the end of the clamp (3 samples per condition; 12 samples total), were diluted to 100 ng/ μl and used to perform the target preparation using the Whole Transcript Sense Target Labeling Protocol procedure (Affymetrix, High Wycombe, UK). 5.5 μg of each fragmented cDNA were end-labeled with biotin and hybridized to a GeneChip Mouse Gene 1.0 ST array (Affymetrix), then processed and scanned according to standard procedures. Background subtraction, quantile normalization and probeset summarization was performed with the *Affymetrix Power Tools* command line tool, using the RNA method [18]. All subsequent statistical analyses were performed using R (http://www.R-project.org) and Bioconductor packages (http://www.Bioconductor.org). Differential hybridized features were identified using Bayes moderated t statistics with the Bioconductor package "limma" [19]. Genes with a p-value < = 0.01 (unadjusted) were considered here as differentially expressed. Hierarchical clustering of samples and differentially expressed genes was performed on matrices of sample or gene distances (Euclidean), using Pearson correlation with average-linkage clustering. Heatmaps were generated using the *gplots* package of Bioconductor. All gene expression data is MIAME compliant and the raw data has been deposited in a MIAME compliant database (GEO accession numbers GSE67523)

### Gene Set Enrichment Analysis (GSEA)

Pathway analysis was performed against mouse MSigDB canonical pathways (V3) using the GSEA algorithm [20]. Genes were ranked by fold change difference (high to low) between hypoglycemia and euglycemia at 48 hours prior to perfoming GSEA. The resulting p values were corrected for multiple comparisons with Benjamini-Hochberg correction in R. The adjusted p-value was used to filter the pathways enriched for up- and down-regulated genes: pathways enriched with an adjusted p value < = 0.05 were kept for further analysis.

### Terminal dUTP Nick End-Labeling (TUNEL) of fragmented DNA

*In situ* cell death detection was performed 48 h after hypoglycemic clamp, by TUNEL technology as described by the manufacturer (Roche Applied Science, Rotkreus, Switzerland) and detailed in Hamann S. *et al*. [10]. For each condition, apoptotic cells were visualized under a fluorescence microscope (Leica) using appropriate filters.

### Immunostaining

Enucleated eyes were fixed in 4% PFA/PBS for 45 min, followed by cryoprotection in 30% sucrose/PBS. Ten μm-embedded frozen sections were further processed for immunohistochemistry. Briefly, frozen retina sections were blocked in PBS with 3% normal goat serum (Sigma, Buchs, Switzerland) and 0.2% Triton X-100 (Sigma) for 1 h at RT and then incubated with antibodies against cleaved Caspase3 (dilution 1/500, Cell Signaling Technology, Inc. Danvers, MA, USA), GPX3 (dilution 1/50, Abcam, Cambridge, MA, USA), GSTO-1 (dilution 1/200, Santa-Cruz Biotechnology, Dallas, TX, USA), FIBULIN-1 (dilution 1/200, Santa-Cruz Biotechnology, Dallas, TX, USA) and VERSICAN (dilution 1/100, Abcam, Cambridge, MA, USA) in the blocking buffer overnight at 4°C. Thereafter, sections were incubated again in blocking buffer for 30 min at RT before being incubated for 1 h at RT with FITC Alexa-Fluor 594 goat anti-rabbit antibody or with FITC Alexa-Fluor 594 sheep anti-goat antibody (dilution 1/2’000,) depending of primary antibody used. Incubation with secondary antibody alone was used as a negative control. Tissue sections were counterstained with DAPI to identify retinal cell layers.

### GSH content analysis

GSH content was measured as previously described [3], briefly twenty μg of total protein lysates, isolated from the retina of single hypoglycemic (sHypo), multiple hypoglycemic (mHypo), single euglycemic (sEugly) and multiple euglycemic (mEugly) mice, sacrificed 48 h post-clamp, were used for the measurement of GSH retinal content. The GSH content was measured with Bioxytech GSH/GSSG-412 kit (#21040; OxisResearch, Beverly Hills, CA, USA) according to the protocol.

### RT-PCR analysis

Eight hundred ng of total RNA in a 20 μl reaction were used for cDNA synthesis using oligo (dT)18 according to the manufacturer’s procedure (First strand cDNA synthesis kit for RT-PCR, Roche Applied Science, Rotkreuz, Switzerland). The equivalent of 2 to 20 ng original total RNA was used for quantitative PCR amplification using the 2 x brilliant SYBR Green QPCR Master Mix (Stratagene) and 0.5 μM forward and reverse primer pair, designed to span an intron of the target gene. Real-time PCR was performed in multiple replicate in the Mx3000PTM system (Stratagene) with conditions previously described [3].

### Statistical Analysis

All results were expressed as means ± SEM of the indicated number of experiments. Data were statistically analyzed using Prism 6.0 software. We first tested each group of data for distribution normality using Shapiro-Wilk tests. In case of normal distribution, we used a Welch’s ANOVA test (one-way ANOVA with unequal variances) followed by a post-hoc Tukey-Kramer test to compare the different treatments. When the distribution was not normal, we used a Kruskal-Wallis test (non-parametric analogue of the one-way ANOVA) to compare the different treatments. p < 0.05 was chosen as the threshold for statistical significance.

## Results

### Insulin-induced hypoglycemia regulates gene expression

**Fig 1:**
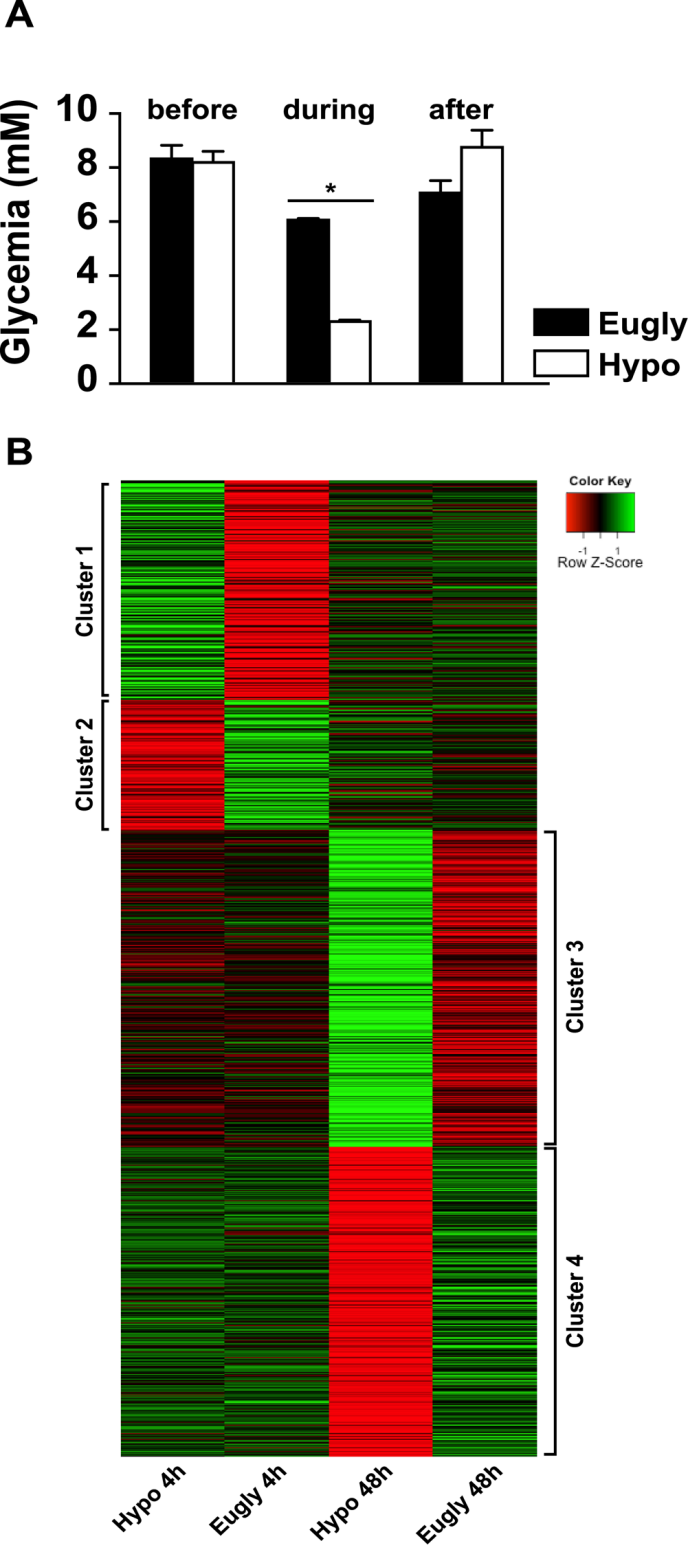
Identification of differentially expressed genes during hyperinsulinemic/hypoglycemic clamp. A) Mouse plasma glucose concentration before, during and after the clamp. *p<0.0001 Hypo (n = 18) vs. Eugly (n = 16). B) Heatmap of significantly differentially regulated genes (p≤0.01) at 4 hours and 48 hours, showing mean normalized intensities (n = 3) at each time-point and condition.

We performed a single hyperinsulinemic/hypoglycemic clamp on 18 different mice after a short period of fasting; hyperinsulinemic/euglycemic clamp was used as controls (16 mice). Glycemia before the clamp was similar in both groups of mice and clearly different during the 4-hours of the clamp (Fig 1A). We isolated three retinas from each group of mice, 4 h and 48 h after the end of the clamp, and extracted mRNA for microarray analysis. Fig 1B shows a heat map with the visualization of four different clusters of genes up-regulated (cluster 1) or down-regulated (cluster 2) 4 h after the clamp, up-regulated (cluster 3) or down-regulated (cluster 4) 48 h after the clamp. Tables 1 and 2 show the top 15 genes up- or down-regulated in each cluster.

**Table 1:** Top 15 genes up/down-regulated by hypoglycemia ranked by fold change (FC) tested 4 hours after the hyperinsulinemic/hypoglycemic clamp.

Symbol	Gene Name	FC	p value	Gene ID	Aff. ID
**Top 15 genes up-regulated by hypoglycemia ranked by fold change (FC)**					
Kif4	Kinesin family member 4	3.4	2.5E-6	16571	10601011
Gas5	Growth arrest specific 5	2.2	8.5E-5	14455	10351039
Etnppl	Ethanolamine-Phosphate Phospho-Lyase	2.0	6.1E-5	71760	10496077
Selenbp1	Selenium binding protein 1	1.8	1.1E-3	20341	10494114
Tc2n	Tandem C2 domains, nuclear	1.8	1.8E-3	74413	10402195
Mt2	Metallothionein 2	1.7	2.2E-4	17750	10574023
Actr6	ARP6 actin-related protein 6	1.7	8.5E-5	67019	10371830
Folh1	Folate hydrolase	1.7	6.5E-4	53320	10565401
Pdcd5	Programmed cell death 5	1.6	2.7E-3	56330	10360983
Ccne2	Cyclin E2	1.6	5.1E-4	12448	10503264
Fkbp5	FK506 binding protein 5	1.6	5.0E-4	14229	10449452
Rbm3	RNA binding motif protein 3	1.6	1.9E-5	19652	10556113
Selenbp2	Selenium binding protein 2	1.6	2.3E-4	20342	10494085
Eif3e	Eukariotic translation initiation factor 3, subunit E	1.6	1.9E-3	16341	10428398
Rpl5	Ribosomal protein L5	1.6	9.0E-3	100503670	10459669
**Top 15 genes down-regulated by hypoglycemia ranked by fold change (FC)**					
Fbln1	Fibulin 1	2.0	8.1E-2	14114	10425945
Cryaa	Crystallin, alpha A	1.9	6.0E-2	12954	10443830
Dct	Dopachrome tautomerase	1.7	8.8E-3	13190	10422249
Crygs	Crystallin, gamma S	1.7	2.9E-3	12970	10438668
Olfr715	Olfactory receptor 715	1.6	6.6E-3	258776	10566624
Slc26a4	Solute carrier family 26, member 4	1.6	7.0E-2	23985	10399854
Ldlr	Low density lipoprotein receptor	1.6	1.6E-5	16835	10583732
Olfr767	Olfactory receptor 767	1.6	1.3E-4	258315	10373610
Gja1	Gap junction protein, alpha 1	1.6	1.2E-1	14609	10363173
Cryba4	Crystallin, beta A4	1.6	6.9E-4	12959	10532517
Tek	Endothelial-specific receptor tyrosine kinase	1.6	4.0E-4	21687	10505954
Fmod	Fibromodulin	1.6	3.4E-2	14264	10349947
Tmem132b	Transmembrane protein 132B	1.6	5.0E-4	208151	10525923
Pltp	Phospholipid transfer protein	1.5	1.0E-2	18830	10489569
Cryba1	Crystallin, beta A1	1.5	1.7E-1	12957	10388707

FC (Fold Change) in gene expression 4h after the hypoglycemic clamp, euglycemic clamp is used as control

**Table 2:** Top 15 genes up/down-regulated by hypoglycemia ranked by fold change (FC) tested 48 hours after the hyperinsulinemic/hypoglycemic clamp.

Symbol	Gene Name	FC	p Value	Gene ID	Aff. ID
**Top 15 genes up-regulated by hypoglycemia ranked by fold change (FC)**					
Gpx3	Glutathione peroxidase 3	4.8	1.2E-2	14778	10376201
Fbln1	Fibulin 1	4.5	4.9E-3	14114	10425945
Slc26a4	Solute carrier family 26, member 4	4.3	1.8E-3	23985	10399854
Klk1b22	Kallikrein 1-related peptidase b22	4.0	5.0E-2	13646	10552594
Penk	Preproenkephalin	3.8	2.0E-2	18619	10511363
Gja1	Gap junction protein, alpha 1	3.4	2.8E-3	14609	10363173
Vcan	Versican	3.0	1.4E-2	13003	10410931
Hba-a2	Hemoglobin alpha, adult chain2	2.7	1.2E-4	110257	10375058
Hba-a1	Hemoglobin alpha, adult chain 1	2.7	1.1E-4	15122	10375051
Atp1a2	ATPase, NA^+^/K^+^ transporting, alpha 2 polypeptide	2.6	3.7E-3	98660	10360270
Slc6a13	Solute carrier family 6, member 13	2.5	2.9E-3	14412	10541318
Serpina3n	Serine peptidase inhibitor, clade A, member 3N	2.4	4.4E-4	20716	10398075
Fmod	Fibromodulin	2.4	8.9E-3	14264	10349947
Krt5	Keratin 5	2.3	1.3E-3	110308	10432785
Optc	Opticin	2.2	9.7E-3	269120	10357858
**Top 15 genes down-regulated by hypoglycemia ranked by fold change (FC)**					
Gluld1	Glutamate-amoniase ligase (glutamine synthetase)	3.1	2.0E-1	266744	10345190
Crygd	Crystallin, gamma D	2.6	4.1E-1	12967	10355191
Crygc	Crystallin, gamma C	2.6	3.3E-1	12966	10355193
Crygb	Crystallin, gamma B	2.4	3.3E-1	12965	10355199
Bfsp2	Beaded filament structural protein 2, phakinin	2.3	2.7E-1	107993	10596190
Bfsp1	Beaded filament structural protein 1, in lens-CP94	2.1	3.2E-1	12075	10488185
Lim2	Lens intrinsic membrane protein 2	1.7	2.0E-1	233187	10552398
Tgfb3	Transforming growth factor, beta 3	1.7	6.0E-2	21809	10430929
Mpv17	MpV17 mitochondrial inner membrane protein	1.7	1.3E-2	17527	10529091
Adam4	A disintegrin and metallopeptidase domain 4	1.7	9.0E-2	11498	10401320
Lctl	Lactase-like	1.6	5.0E-2	235435	10586140
Nebl	Nebulette	1.6	1.1E-1	74103	10480286
Psg29	Pregnancy-specific glycoprotein 29	1.6	3.0E-2	114872	10550424
Fabp5	Fatty acid binding protein 5, epidermal	1.6	2.5E-2	16592	10490838
Hspb1	Heat shock protein 1	1.6	3.1E-1	15507	10526410

FC (Fold Change) in gene expression 48h after the hypoglycemic clamp, euglycemic clamp is used as control

**Fig 2:**
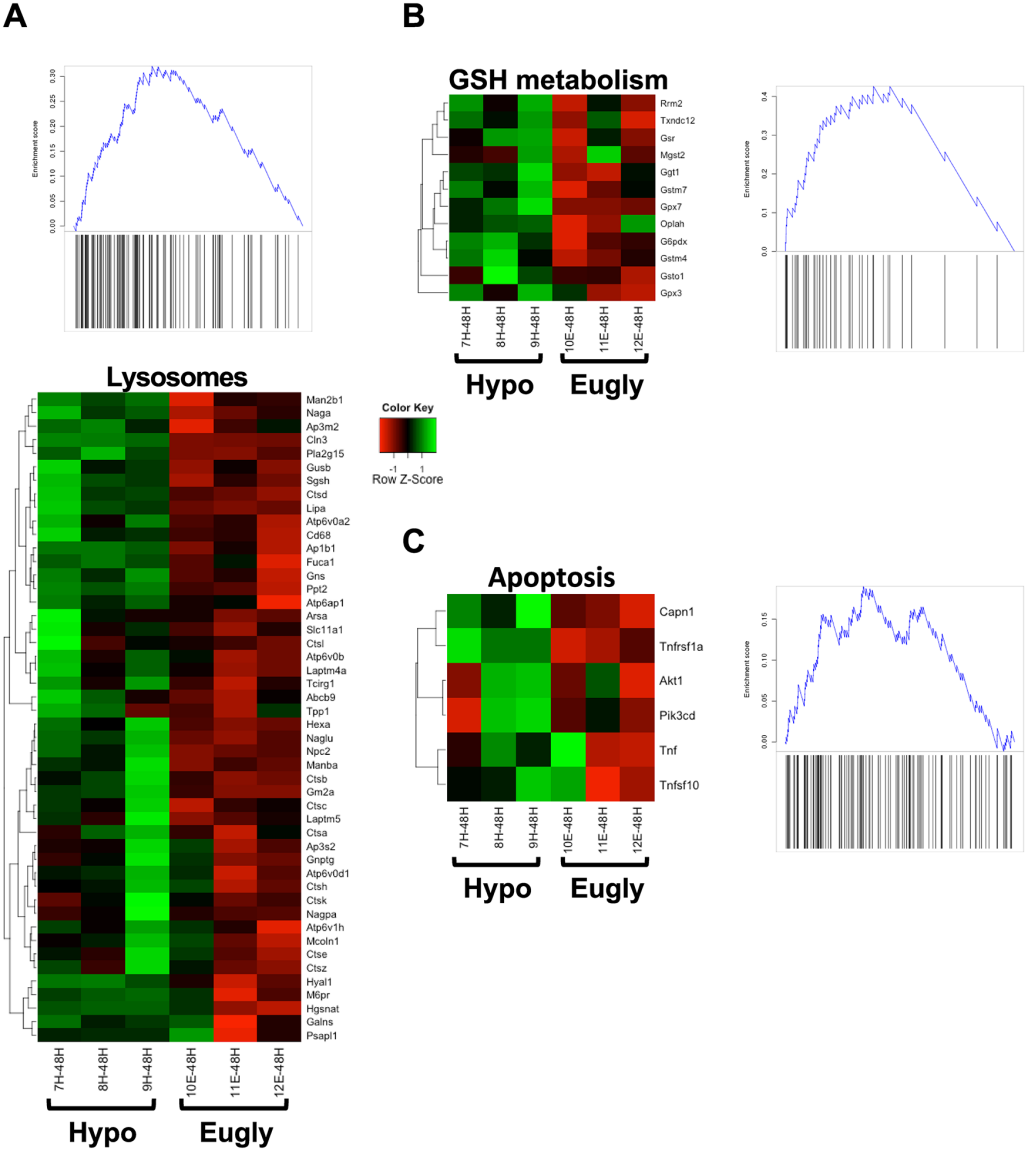
Heatmaps of selected pathways that were significantly enriched for hypoglycemia up-regulated genes at 48 hours identified by GSEA. A) KEGG Lysosome pathway; B) KEGG Glutathione metabolism; C) REACTOME Apoptosis. The genes shown in the heatmaps are the leading edge subsets (the enriched genes) from the GSEA analysis [20]. GSEA score plots are shown alongside the heatmaps: Here a score peak towards the left indicates enrichment of pathway genes at the top of the list of genes ranked by fold-change. All three pathways shown were significantly enriched (corrected p-value ≤ 1e-4) based on 10000 permutations.

We performed a sensitive pathway analysis (Gene Set Enrichment Analysis: GSEA [20]) comparing hypoglycemia vs euglycemia 48 h after a single clamp and we highlighted three important pathways, including KEGG lysosomes (Fig 2A), KEGG GSH metabolism (Fig 2B) and REACTOME-apoptosis (Fig 2C). Moreover, we were able to point out several glycoproteins (Fibulin1, Versican, Fibromodulin) linked to the extracellular matrix (ECM), many crystallins and few solute carriers (Slc26a4, Slc6a13) whose expression was modified by hypoglycemia.

**Fig 3:**
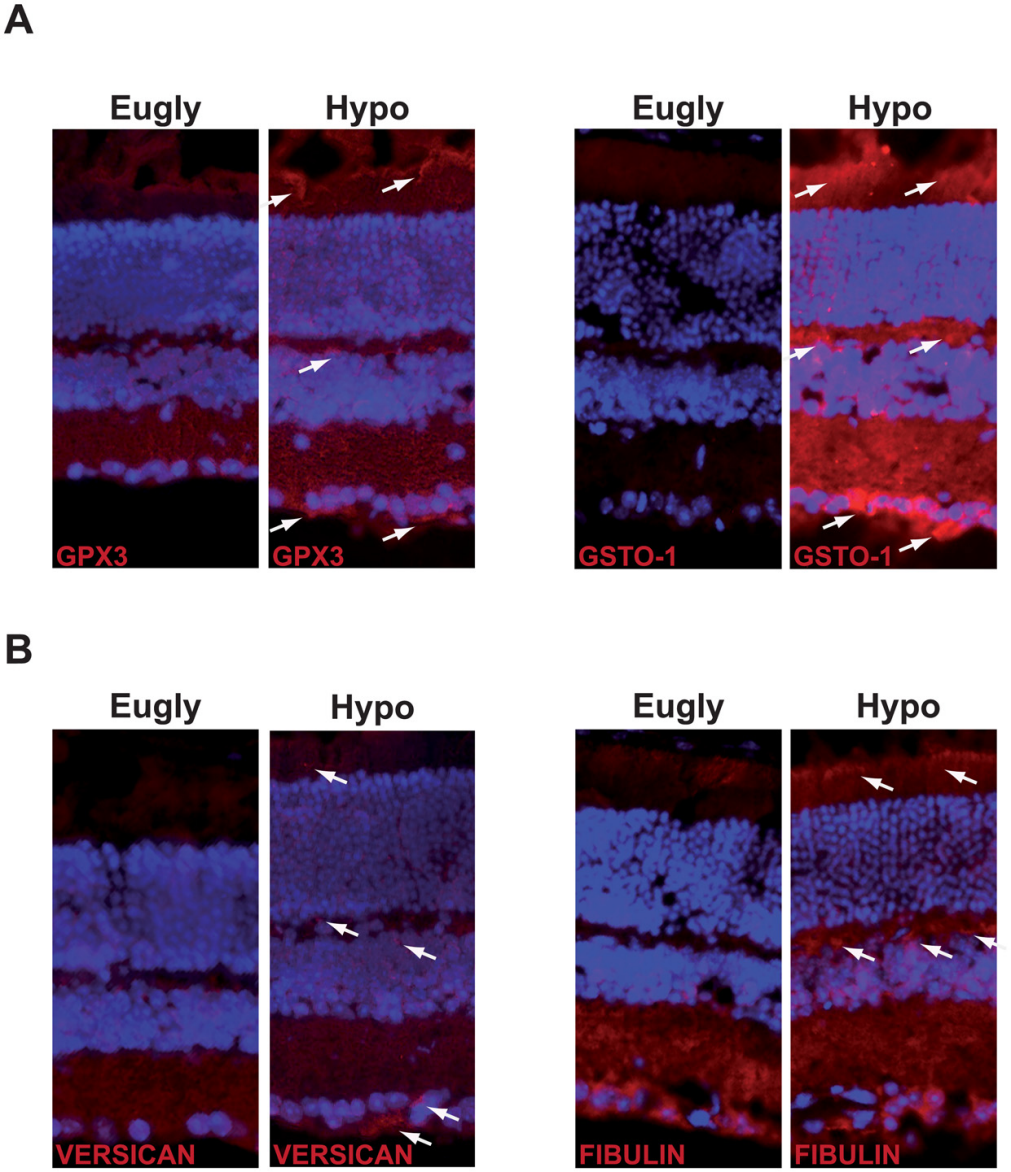
Increase expression of proteins involved in GSH metabolism and in the establisment of the extracellular matrix. Immunostaining of GPX3 and GSTO-1 proteins (A), and VERSICAN and FIBULIN-1 (B) in the retina of mice submitted to a hypoglycemic (Hypo) or euglycemic (Eugly) clamp (white arrows showed accumulation of proteins of interest). This experiment is representative of 3 retinas for each group.

To confirm that genes showing an increase at mRNA level (Table 2), also showed an increase at protein level we performed several immunostaining of proteins involved in GHS metabolism (GPX3 and GSTO-1) and in establishment of the extracellular matrix (VERSICAN and FIBULIN-1). Fig 3 shows an increased immunostaining of GPX3 and GSTO-1 principally in photoreceptor outer segment (POS), inner plexiform layer (IPL) and ganglion cell layer (GCL). Proteins of the extracellular matrix, VERSICAN and FIBULIN-1 show similar staining with a significant increase of FBULIN-1 in POS and IPL, while VERSICAN staining is principally increased in GCL (Fig 3A and 3B).

### Multiple successive acute hypoglycemias did not induce retinal cell death in mouse in comparison with single acute hypoglycemia

**Fig 4:**
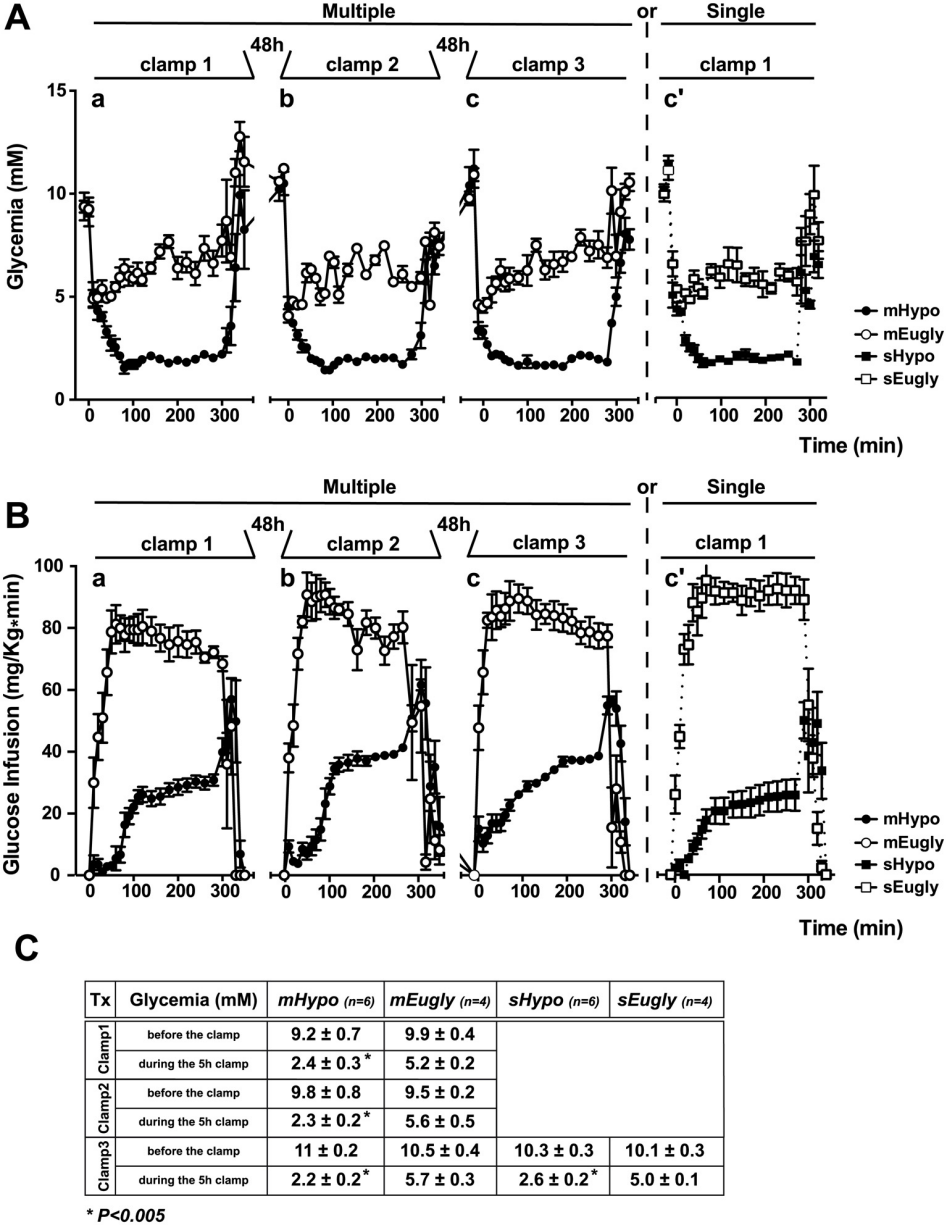
Insulin-induced multiple and single hypoglycemia in C57BL/6 mouse. A) Graphic representation of plasma glucose levels during hyperinsulinemic clamps. Three successive hypoglycemic (mHypo; n = 6) and euglycemic (mEugly; n = 4) clamps (right part of the graph) spaced by 48 h were performed; during the last clamp, single hypoglycemic (sHypo; n = 6) and euglycemic (sEugly; n = 4) clamps were performed additionally (left part of the graph). B) Graphic representation of glucose infusion rates during all hyperinsulinemic/hypoglycemic clamps (black circle for mHypo and black square for sHypo) and the control hyperinsulinemic/euglycemic clamp (white circle for mEugly and white square for sEugly). Multiple clamps are showed in the left part of the graph, while single clamps are showed in the right par of the graph C) Mouse characteristics before and during the clamp. *p<0.005 Hypo vs. Eugly.

We recently showed that acute (4 h) hypoglycemia strongly induces retinal cell death in mice. In order to analyze the effect of multiple successive hypoglycemia on mouse retina, we performed three hyperinsulinemic/hypoglycemic clamps (mHypo), spaced by 48 h each. We performed three similar hyperinsulinemic/euglycemic clamps (mEugly) as control group. In parallel with the last clamp, we added two groups including a single hyperinsulinemic/hypoglycemic clamp (sHypo) as positive control and a single hyperinsulinemic/euglycemic clamp (sEugly) as negative control. Both euglycemic groups (mEugly and sEugly) of mice received comparable amounts of insulin but were injected with glucose in order to maintain normal glycemia. Comparison of these four groups allowed us to analyze the effect of multiple successive hypoglycemias in mice. We monitored glycemia throughout all clamps (Fig 4A). Glucose infusion, necessary to maintain either the hypoglycemia at 2.2 mM or the euglycemia at 5–6 mM, is shown in fig 4B. Glycemia before the clamp was similar for both groups, while a significant expected difference in glycemia was observed during the clamp (Fig 4C). We observed that glucose infusion rate necessary to maintain hypoglycemia around 2.2 to 2.4mM was more important for the second and third clamp in comparison to the first clamp or to the single hyperinsulinemic/hypoglycemic clamp (S1 Fig). Indeed, we injected less glucose during the first (23.167 ± 4,74 mg*Kg^−1^*min^−1^) and the single clamp (22.25 ± 6.8 mg*Kg^−1^*min^−1^) in comparison to the second (37.5 ± 1.87 mg*Kg^−1^*min^−1^) and the third clamp (36.6 ± 0.99 mg*Kg^−1^*min^−1^).

**Fig 5:**
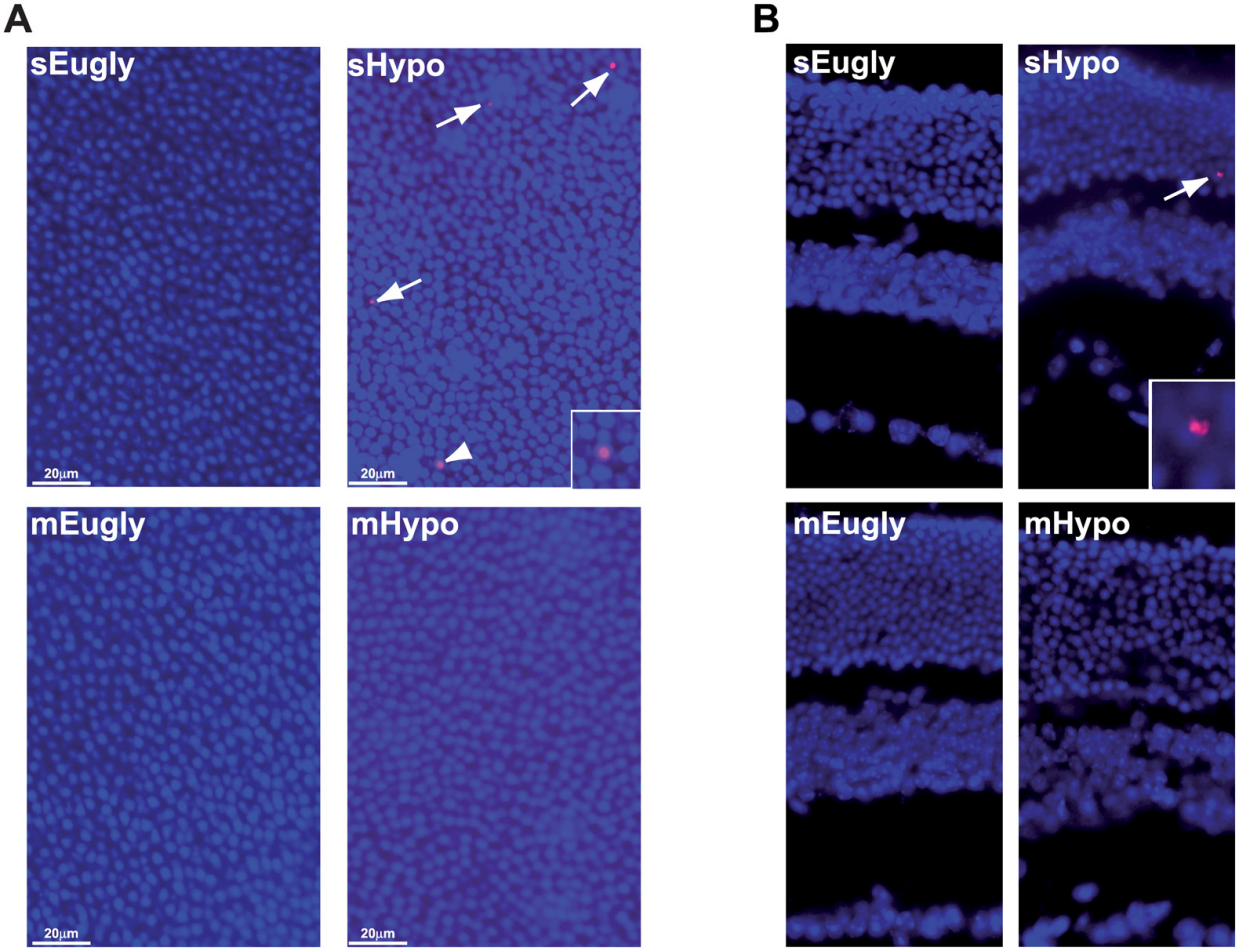
Multiple successive hypoglycemias did not exacerbate cell death in mouse retina but protect against retinal apoptosis. A) Flat-mounted retinas were isolated 48 h after the clamp, stained for cell death by TUNEL assay and DAPI counter coloration was performed. White arrows show TUNEL positive cells in single hypoglycemic condition (sHypo) while no TUNEL positive cells were detected when multiple successive hypoglycemia (mHypo) was applied. Control situation with multiple euglycemia (mEugly) or single euglycemia (sEugly) showed, as expected, no TUNEL positive cells. Results were representative of two (sEugly/mEugly) to three (sHypo/mHypo) observed flat-mount retinas for each group. B) Cleaved Caspase 3 antibody showed positive cells (white arrow) in the outer nuclear (ONL) of the hypoglycemic mice retina, while no positive cells were observed in all other conditions. Counterstaining with DAPI was performed to identify the retinal cell layers. Results were representative of four (sEugly), one (mEugly), four (sHypo) or two (mHypo) observed retinas for each group.

We then isolated flat-mount retinas 48 h after the clamp, and performed TUNEL staining in each group. Fig 5A shows TUNEL-positive cells only in the retina from mice, which experienced a single hypoglycemic event. Very few positive cells, if any, were observed in multiple successive hypoglycemic (mHypo) and in both euglycemic conditions (mEugly and sEugly) and control sham-operated mice (data not shown).

Apoptosis is often associated with caspase 3 activity in diverse models, including hyperinsulinemic/hypoglycemic clamp (sHypo) that we previously described [3]. Interestingly, the same as no retinal cell death observed after three successive hypoglycemic events (Fig 5A), neither were we able to observe any Caspase 3 activation in those retinas. Fig 5B shows positive cells in the ONL of mice suffering a single hypoglycemic event, while no positive cells for cleaved caspase 3 were observed in the retina of the other groups of mice (mHypo, mEugly and sEugly).

### Reduced glutathione (GSH) content played key role in cell death

**Fig 6:**
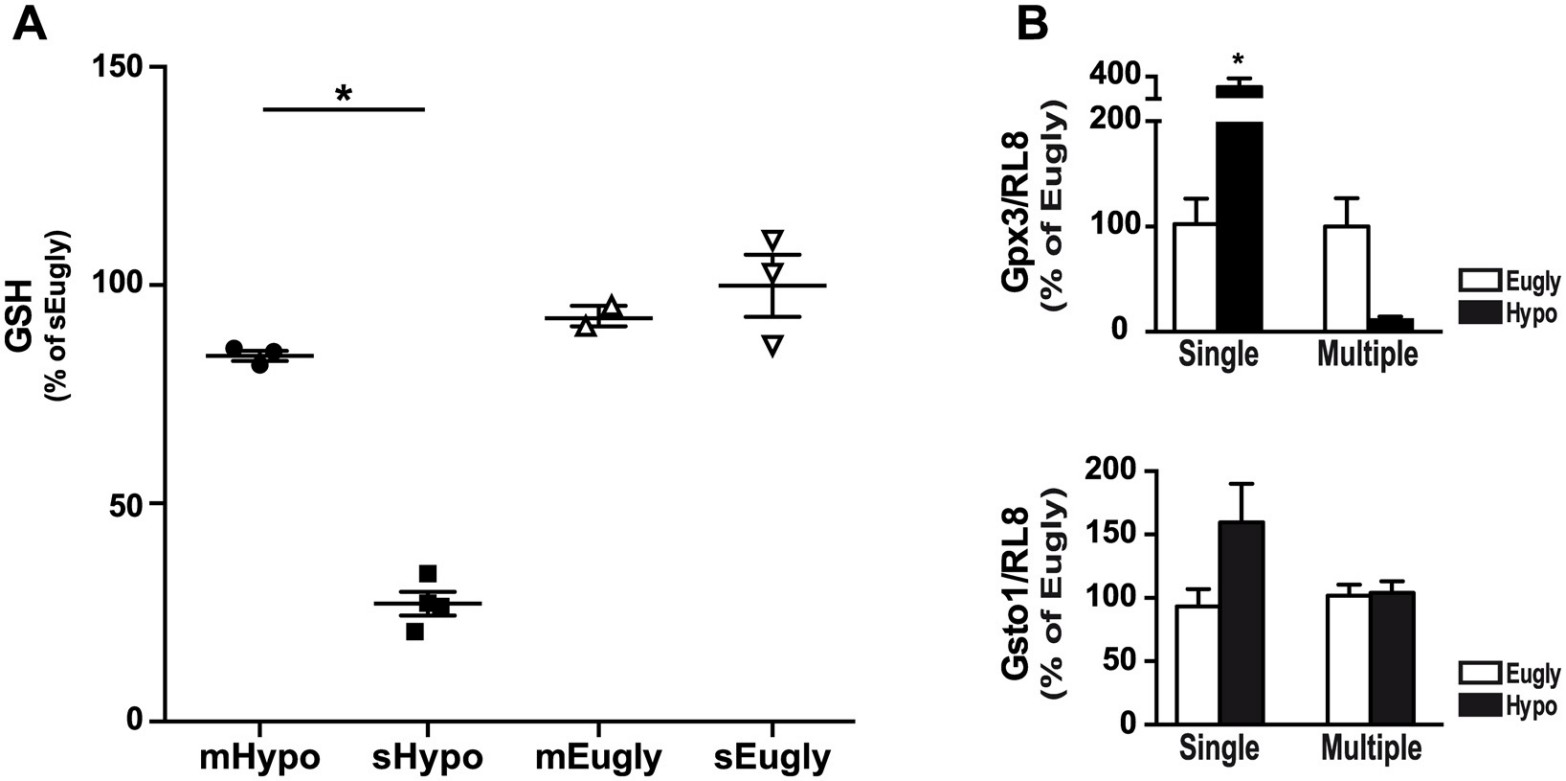
GSH homeostasis was modulated in the retina of mice suffered single hypoglycemia but not after multiple successive hypoglycemic events. A) GSH content was measured in protein lysates obtained from retina of single euglycemic (sEugly: white inverted triangle), multiple euglycemic (mEugly: white triangle), single hypoglycemic (sHypo: black square) and multiple hypoglycemic (mHypo: black circle) mice. Results were expressed as percent of sEugly and as mean ± SEM of 2 to 4 samples. *p<0.01 sHypo vs. sEugly. B) We tested the expression of two genes, Gpx3 and Gsto1, in the retina of hypoglycemic and euglycemic animals 48 h after the clamp. We were able to show, by RT-qPCR, the induction of both genes in single hypoglycemic conditions, but not after multiple successive hypoglycemic or euglycemic. *RL8* (ribosomal protein L8) was used as internal control to normalize RNA expression and results are expressed as percent of respective control (sEugly, mEugly) and as mean ± SEM of 3 retinas, *p<0.04.

We recently showed the key role of GSH in retinal cell death. Indeed, GSH metabolism is altered as observed in pathway analysis (Fig 2B) and GSH content was decreased when mice suffered a single hypoglycemic event (this study (Fig 6A) and [3]). When we tested the GSH level in all groups of mice, we only observed, as expected, a marked decrease of GSH in mice suffering a single hypoglycemia, while multiple successive hypoglycemic events did not alter GSH content, or restore normal GSH level. Control groups, single and multiple euglycemia, also showed no modification of GSH content (Fig 6A). Interestingly, we previously showed that GSH metabolism is decreased during hypoglycemia, with both an increase of the glutathione peroxidase 3 (Gpx3) and the glutathione S-transferase omega 1 (Gsto-1) expression either in vivo during single hypoglycemia, and in 661W photoreceptor cells or in C57BL/6 isolated retina cultured at low glucose concentration [3]. As expected and showed in fig 6B, we observed an increase in the expression of Gsto-1 and Gpx3 expression only after a single hypoglycemia, while mRNA expression of Gsto-1 was not changed after multiple successive hypoglycemic clamps and neither was Gpx3 obviously decreased (Fig 6B).

## Discussion

The sequential microarray analysis of mice suffering hypoglycemic events revealed multiple modifications in retinal gene expression, either 4 h or 48 h after the clamp. Interestingly, we highlight broad changes in many different pathways, including GSH metabolism, lysosomal regulation and apoptosis. Indeed, we confirmed our previous results showing the key role of GSH in low glucose-induced retinal cell death [3]. We have previously shown by qPCR, the increase of Gpx3 (the top gene up-regulated by hypoglycemia; fold change = 4.8), and Gsto-1 (up-regulated by hypoglycemia; fold change = 1.9) in comparison with euglycemic control situation. Genetic expression of both genes was also increased when isolated mouse retinas were cultured 48 h at low glucose condition (Gpx3, fold change = 5 and Gsto-1, fold change = 1.5) [3]. Interestingly, microarray analysis also pointed out the down-regulation of the glutamine synthetase (Gluld1), also called Legsin, a protein highly expressed in lens with strong similarity to glutamine synthetase type I (top gene down-regulated by hypoglycemia; fold change = 3.1). The role of this protein, as well as its expression pattern in retina, is controversial. Whereas Barragan *et al*. suggest a potential role of LEGSIN (LGS) in glutamate uptake and transport [21], Grassi *et al*. rule out any involvement of the splice variant Lgs_ ν2 in glutamine biosynthesis. Grassi et al. further claim that LGS is preferentially (if not exclusively) expressed in the lens [22]. As we were able to measure LGS expression in retinal mRNA preparations, which are essentially devoid of contamination by, lens mRNA, (S2 Fig), we cannot completely exclude a bona fide expression of Lgs (or another splice variant of Lgs) in the retina. In line with a non-exclusive retinal expression of Lgs, Nakatsugawa *et al*., recently showed that Lgs expression is not restricted to the lens but also found in liver, placenta, skeletal muscle and pancreas. In this latter study the authors also described a novel splice variant of Lgs (Lgs_ν4) expressed in lung [23].

Overall, our present and previous studies provide several lines of evidence supporting a key role of GSH in hypoglycemia-induced cell death, including the up-regulation of Gpx3 and Gsto-1 expression at both mRNA and protein levels (see [3] and Fig 6), the alteration in GSH metabolism suggested by the GSAE analysis (Fig 2) and the demonstration of a causal role of GSH content modulation on retinal cell death [3]. Accordingly, recurrent hypoglycemia leads to the restoration of “normal” GSH level, which correlates with an absence of retinal cell death (Fig 5).

In addition, in our last study we deciphered the mechanism of apoptosis induced by low glucose and suggested a defect of fusion between lysosomes and autophagosomes, probably due to a significant decrease of Lamp2a expression, that led to autophagy inhibition and cell death [4]. Even if Lamp2a expression was not altered in the microarray analysis, we cannot exclude a role of this gene in the process. Moreover, in this study, microarray analysis emphasized both apoptotic and lysosomal pathways that are altered by hypoglycemia.

Microarray analysis suggests a potential role of many proteins that are highly glycosylated and linked to the extracellular matrix (ECM), including glycoproteins (Fibulin1:Fbln1) and proteoglycans (Versican:Vcan, Fibromodulin:Fmod). We observed a decrease of Fbln1 and Fmod 4 h after the clamp, but an increase of both gene expressions as well as an increase of Vcan expression 48 h after the clamp. Both FBLN-1 and VSC proteins were also increase in mice undergoing hypoglycemia. Whereas no specific role for Fbln1 has been described so far in the retina, Fibulin2 (Fbln2) has been implicated in retinal detachment observed in diabetic retinopathy, retinopathy of prematurity and other retinal complications [24]. Fibulin3 (Fbln3) has also been associated with both Malattia Leventinese and Doyne honeycomb retinal dystrophy [25]. Interestingly, Fbln1 has a high affinity for Vcan (also increased in the microarray analysis), this latter protein interacting also with numerous other ECM components such as hyaluronan, type I collagen, fibrillin-1, fibronectin and chemokines [26]. The retinal role of the proteoglycan Fmod is not clear yet, but it has been described to promote angiogenic environment, probably via transforming growth factor beta 1 (TGFb1) secretion; it has also been linked to an enhancement of vascular sprouting during normal retinal development [27]. Abnormal expression of these glycosylated proteins may affect the structure and the neovascularization of the retina via modification of ECM of the retina.

Crystallins are water soluble proteins essentially found in the lens and cornea of the eye. We observed a decrease of alphaA, gammaS, betaA4 and betaA1 4 h after the hypoglycemic clamp (Table 1), while crystallins gammaB, gammaC and gammaD were down regulated 48 h after the clamp (Table 2). Although the decrease of these genes, observed 48h after the clamp, were not significantly differentially expressed according to our criteria, the fact that we see several of them down regulate, 4h after the clamp and few others 48h after the clamp, suggests a possible role for these proteins in the process. Alpha A crystallin has already been implicated in numerous cellular processes, including oxidative stress, neuroprotection, survival and cell death pathways [10, 28], but little is known about the role of the other crystallins that could not only be restricted to the lens but also implicated in a whole gene network in the mouse retina [29].

Microarray analysis also highlights the decrease in the expression of two solute carriers, Slc26a4, (reduced 4 h and 48 h after the clamp) and Slc6a13, (reduced 48 h after the clamp). Slc26 genes family is multifunctional anion exchangers while Slc6 genes family have been described as neurotransmitter symporters, but nothing is known about their potential role in the retina. Even if very recently, another solute carrier, Slc24a1 has been implicated in congenital stationary night blindness [30], further experiments will be needed to clearly implicate those transporters in hypoglycemic-induced retinal cell death and diabetic retinopathy.

When we tested the effect of recurrent hypoglycemia (three successive hypoglycemia, spaced by 48 h), we first observed the need of higher glucose infusion rate in the second and third clamps in comparison to the first and to a single hypoglycemic clamp (S1 Fig). This result supports the hypothesis of a decrease of counterregulatory response to hypoglycemia and impairment to recognize it (HAAF concept), but we cannot exclude that could be at least partially due to a decrease in handling stress response. However, we were surprised not to detect any retinal cell death and Caspase3 activation after recurrent hypoglycemic events. This could be partially explained by an increase in the level of lactate, acting as a neuronal energy source [31] and as a “metabolic regulator” [32]. In fact, both glucose and lactate metabolism adapt during recurrent hypoglycemia and this will preserve normal neuron functions under hypoglycemia [32]. Moreover, lactate could directly enter the Krebs cycle and prevent glutamate, glutamine, and GABA depletion that occur during hypoglycemia or aglycemia [14]. The restoration of “normal” GSH level could partially be explained by the presence of these compounds essential for GSH synthesis; in addition, production of NADPH, which is rate limiting for glutathione reductase activity, could be responsible for the GSH level we observed in recurrent hypoglycemia. We also observed that both Gpx3 and Gsto1 enzymes, which were up-regulated during a single hypoglycemic clamp, were indeed down-regulated (Gpx3) or not changed (Gsto1) during recurrent hypoglycemia (Fig 6). The absence of retinal cell death during recurrent hypoglycemia does not per se a demonstration that hypoglycemic events are harmless or that strict glycemia control could be neglected, because the model of recurrent hypoglycemia, used in this study, may not strictly reflect what occurs in diabetic patients during HAAF.

We hypothesized, however, that GSH level is the key element in the prevention of hypoglycemia-induced retinal cell death; low level of GSH will increase the risk of apoptosis, while “normal” level will prevent cell death. This is supported by numerous studies performed in the brain [33] and by our previous experiments performed on 661W photoreceptor cells where the decrease of GSH level, by buthionine sulphoximine (BSO), induced cell death, while restoration of GSH level at low glucose, by GSH ethyl ester, prevented cell death [3]. Treatment with anti-oxidants, to prevent GSH depletion, may represent an important therapeutic axis to protect retinal cell death in diabetic patients suffering hypoglycemic events.

## Supporting Information

S1 ARRIVE Guidelines Checklist NC3Rs ARRIVE Guidelines Checklist. (PDF)

S1 Fig Glucose infusion (GINF) necessary to maintain hypoglycemia during clamps. Comparison of glucose infusion during hyperinsulinemic/hypoglycemic clamps. (circles for mHypo and square for sHypo). We clearly see an increase of glucose infusion in clamp 2 and 3 (mHypo) in order to maintain hypoglycemia around 2.2 to 2.4mM.
(TIF)

S2 Fig PCR analysis showing the low level of possible cross-contamination of retinal mRNA with lens mRNA. Upper panel showed amplification of Crystallin gamma A (Cryga) from two samples of lens mRNA and from retinal mRNA used for microarray analysis; mRNA with no reverse transcriptase (RT-) was used as negative control. We used Gapdh amplification to normalize. Quantitative PCR was performed with these samples in order to quantify the possible level of cross-contamination. Amplification of Cryga was performed using specific Bio-Rad primers (#10025636) while normalization with Gapdh was performed using the following primers (forward: 5'-GAG GCC GGT GCT GAG TAT GT-3' and reverse 5'-GGT GGC AGT GAT GGC ATG GA-3). Standard conditions was used for PCR analysis with annealing at 60°C.
(TIF)

S1 Table Experiments performed and mice utilisation during single clamp experiment. (TIF)

S2 Table Experiments performed and mice utilisation during multiple and single clamps experiment. (TIF)
