# Supplementary figures and images for: Biological Characterization of Gene Response to Insulin-Induced Hypoglycemia in Mouse Retina
Source: PLoS One. 2016 Feb 26;11(2):e0150266. doi: 10.1371/journal.pone.0150266 (PMC4769281; doi:10.1371/journal.pone.0150266)

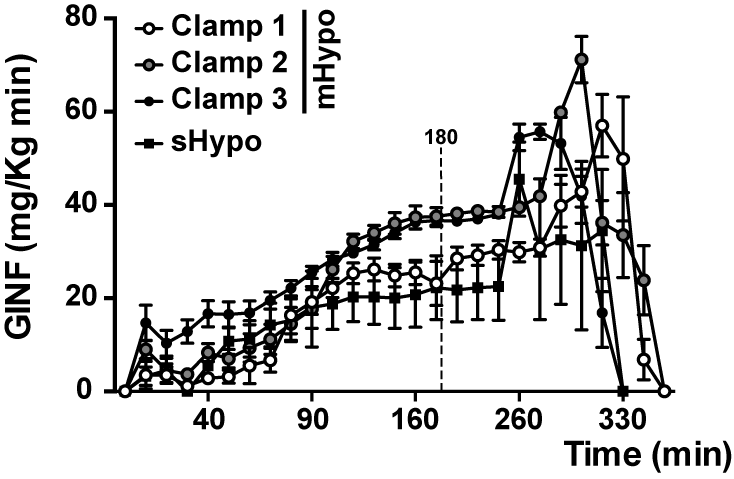

Supplement: S1 Fig — Comparison of glucose infusion during hyperinsulinemic/hypoglycemic clamps. (circles for mHypo and square for sHypo). We clearly see an increase of glucose infusion in clamp 2 and 3 (mHypo) in order to maintain hypoglycemia around 2.2 to 2.4mM. (TIF) [file pone.0150266.s002.tif]

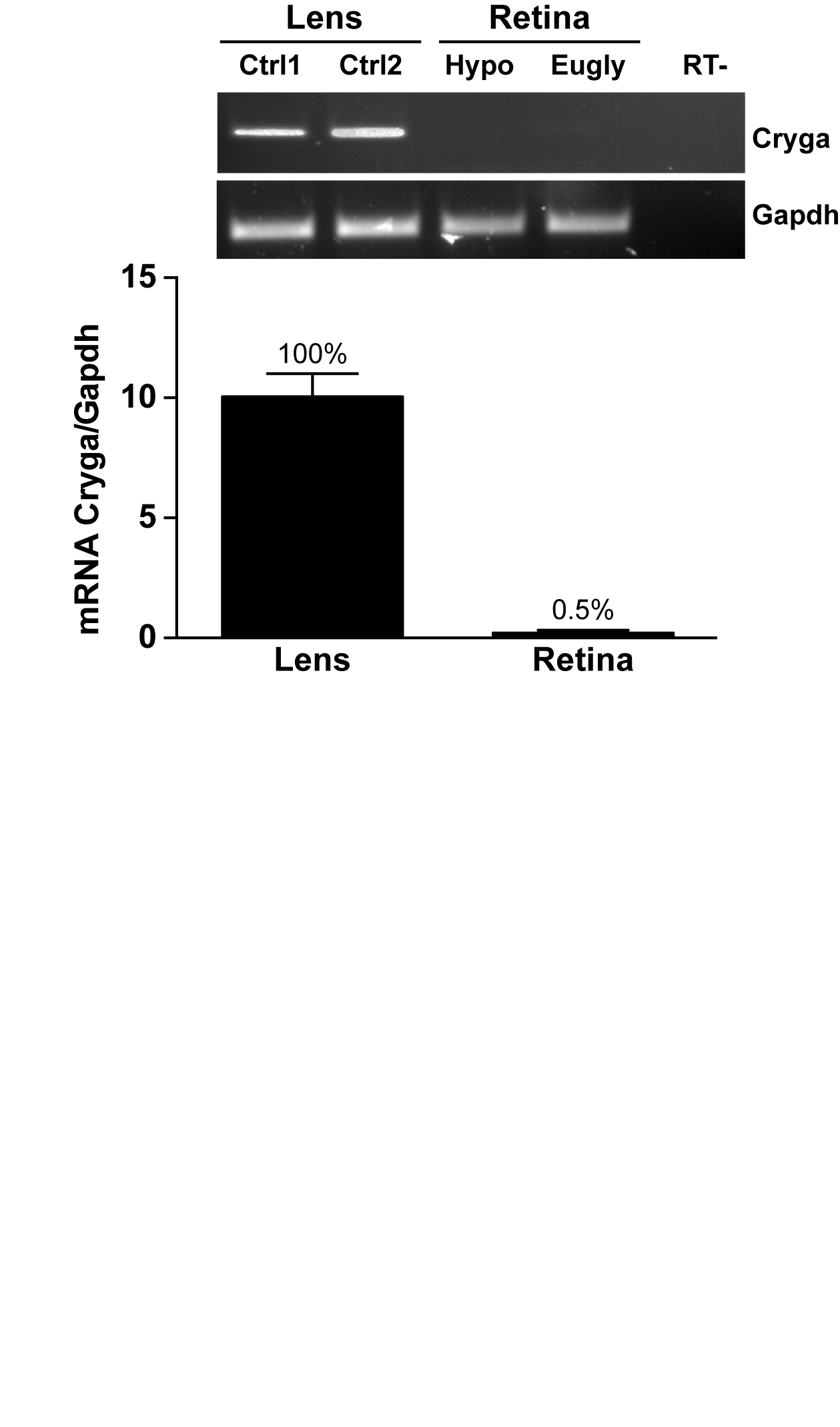

Supplement: S2 Fig — Upper panel showed amplification of Crystallin gamma A (Cryga) from two samples of lens mRNA and from retinal mRNA used for microarray analysis; mRNA with no reverse transcriptase (RT-) was used as negative control. We used Gapdh amplification to normalize. Quantitative PCR was performed with these samples in order to quantify the possible level of cross-contamination. Amplification of Cryga was performed using specific Bio-Rad primers (#10025636) while normalization with Gapdh was performed using the following primers (forward: 5'-GAG GCC GGT GCT GAG TAT GT-3' and reverse 5'-GGT GGC AGT GAT GGC ATG GA-3). Standard conditions was used for PCR analysis with annealing at 60°C. (TIF) [file pone.0150266.s003.tif]

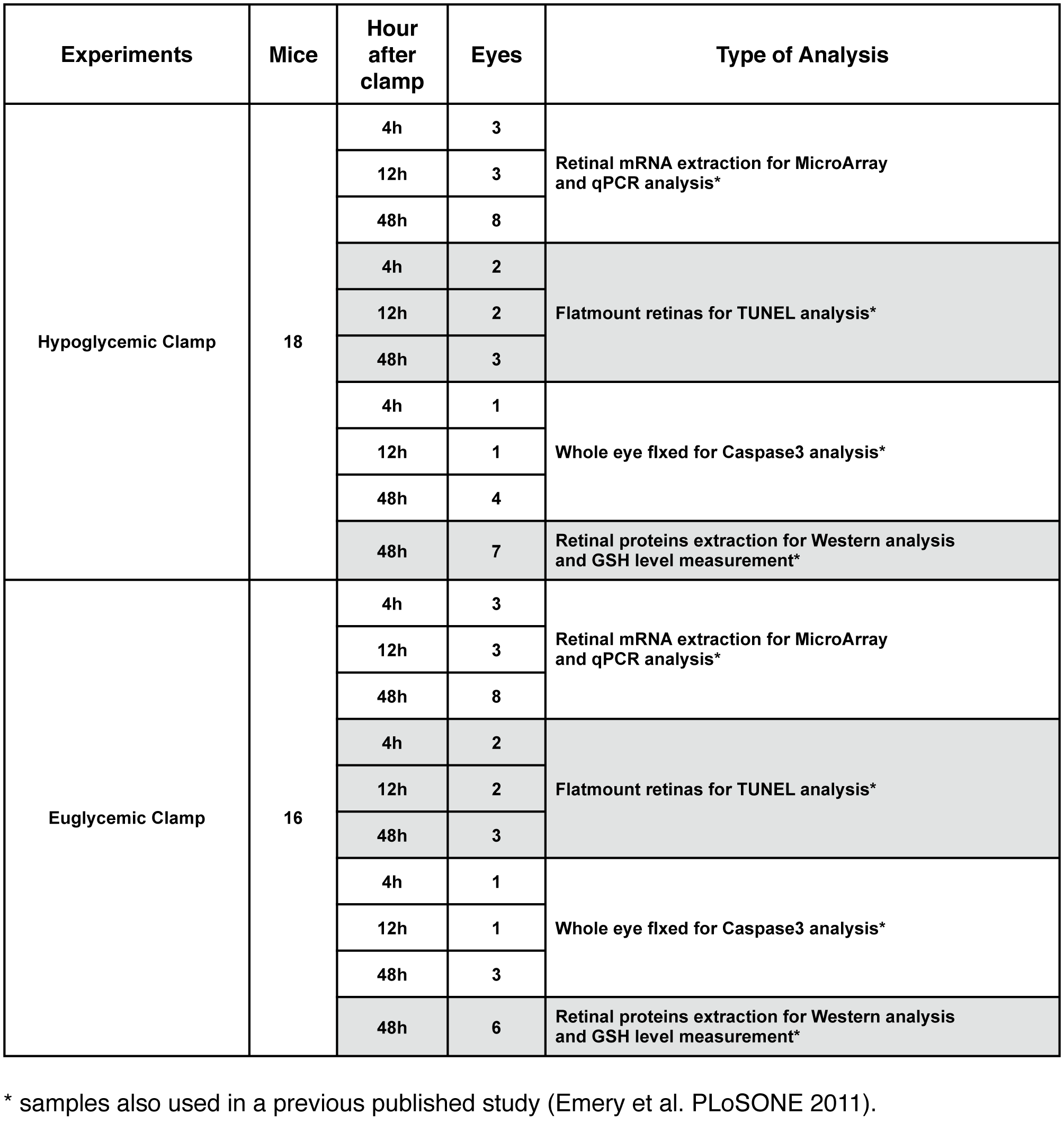

Supplement: S1 Table — (TIF) [file pone.0150266.s004.tif]

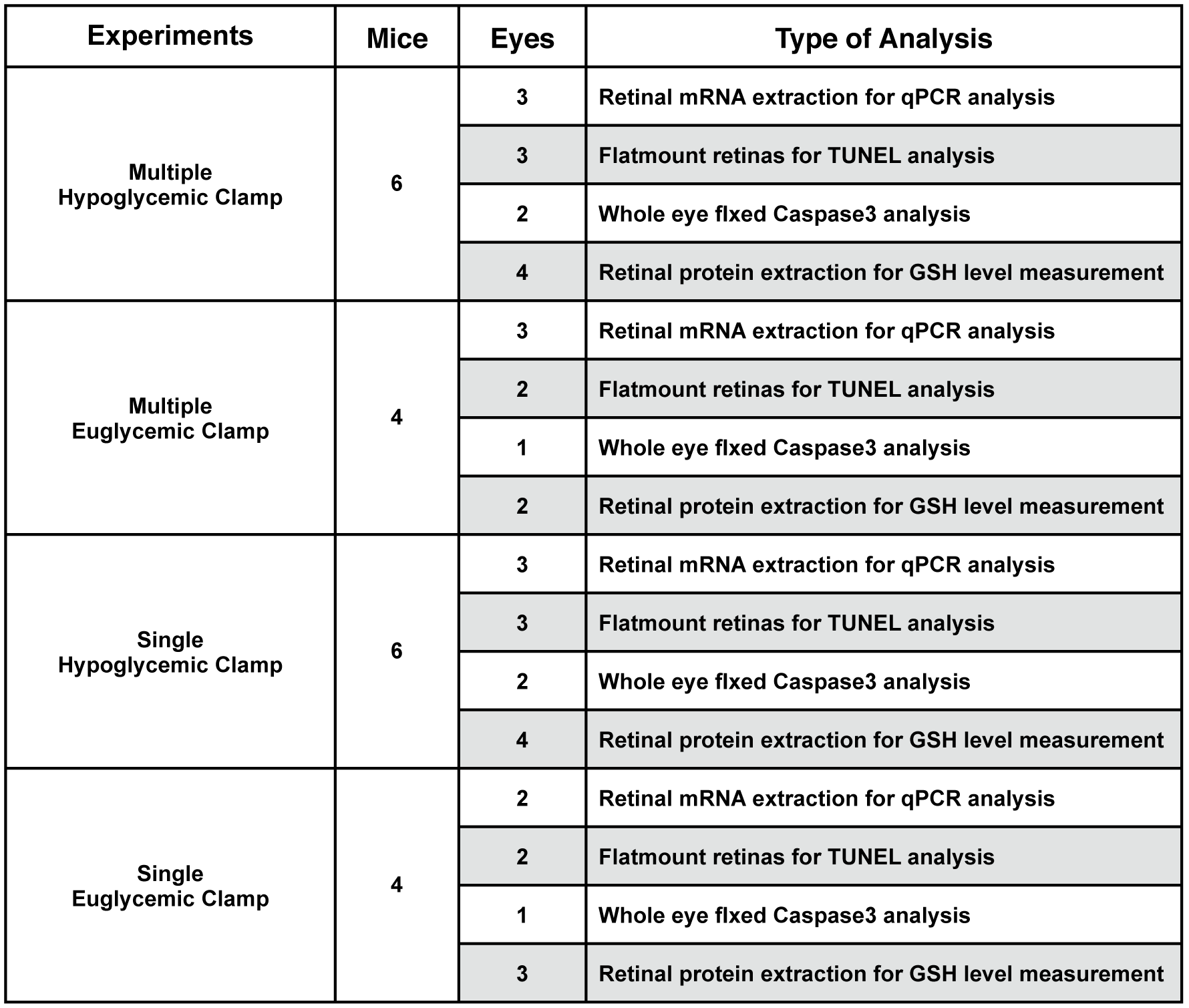

Supplement: S2 Table — (TIF) [file pone.0150266.s005.tif]
